# Post-COVID varicella-zoster virus reactivation: lowering the immunological threshold for latency breakdown

**DOI:** 10.3389/fcimb.2026.1904565

**Published:** 2026-08-05

**Authors:** Xiaolu Li, Shuchang Cheng, Siqi Wang, Kaili Gao, Yang Bai, Sainan Li, Mingchun Gao, Yongli Guo

**Affiliations:** 1 Department of Immunology, School of Basic Medical Sciences, Harbin Medical University, Harbin, Heilongjiang, China; 2 College of Veterinary Medicine, Northeast Agricultural University, Harbin, Heilongjiang, China

**Keywords:** cell-mediated immunity, COVID-19, herpes zoster, immune reserve, latent virus reactivation, long COVID, SARS-CoV-2, varicella-zoster virus

## Abstract

Herpes zoster (HZ) is the clinically apparent manifestation of latent varicella-zoster virus (VZV) reactivation. Aging and immunosuppression are established risk contexts. SARS-CoV-2 infection has prompted renewed attention to whether acute or post-acute immune changes can reduce the reserve needed to maintain VZV latency. Large observational studies report a modest increase in HZ after COVID-19, most consistently after severe disease or hospitalization and within early post-infection windows. These associations do not establish direct causation or a population-wide shift of HZ toward younger adults. A threshold-lowering interpretation is more consistent with the available evidence: SARS-CoV-2 infection may narrow latency-control reserve through cellular immune disruption, interferon dysregulation, and inflammation, particularly in hosts already affected by comorbidity or treatment-related immunosuppression. Long COVID provides a setting in which persistent immune dysregulation can be studied, but it is not yet a proven causal framework for VZV disease. Post-COVID HZ may therefore represent a clinically visible manifestation of disrupted host-virus homeostasis in susceptible individuals. Prospective studies that combine clinical phenotyping with VZV-specific cellular immune measurements are needed to test this model.

## Introduction

Herpes zoster (HZ) results from reactivation of latent varicella-zoster virus (VZV) in sensory and autonomic ganglia, usually when VZV-specific cell-mediated immunity becomes insufficient to contain the virus. Age-related decline in VZV-specific cellular immunity is a central determinant of risk (Levin et al., 2003). Immunosuppression, malignancy, chronic disease, psychological stress, and corticosteroid exposure are additional established risk contexts (Marra et al., 2020). These risk contexts are not interchangeable, but each can reduce the immune reserve that keeps latent infection clinically silent; together, they are consistent with the broader relationship between immunosenescence, loss of functional immune reserve, and HZ in older adults (Curran et al., 2023). HZ therefore provides a useful clinical window into the destabilization of a persistent host-virus equilibrium.

The COVID-19 pandemic placed this host-virus equilibrium in a new clinical context. Early reports of HZ during or after COVID-19 consisted largely of case reports, short clinical series, and ecological comparisons. Later observational analyses included a United States claims cohort (Bhavsar et al., 2022), a global TriNetX cohort (Chen et al., 2023), and a Spanish population cohort (Correcher-Martínez et al., 2024); each reported an association between SARS-CoV-2 infection and HZ. A subsequent meta-analysis synthesized the broader post-infection evidence (Wang et al., 2024), and a Japanese self-controlled study provided complementary evidence within short post-infection windows (Mizuno et al., 2025). The relevant issue is how to interpret these heterogeneous findings. A direct and uniform causal pathway is unlikely. SARS-CoV-2 infection may instead narrow the immune margin required to maintain VZV latency, particularly in hosts whose reserve has already been reduced by age, comorbidity, treatment, or severe systemic inflammation.

This Mini Review focuses on VZV reactivation after SARS-CoV-2 infection rather than the broader spectrum of COVID-19-associated cutaneous disease or vaccine-associated HZ. We integrate epidemiological timing, VZV latency biology, post-acute immune changes, and host modifiers in a threshold model intended to organize the evidence and identify hypotheses for longitudinal testing. The model links clinical observations with broader questions of host-virus homeostasis, immunosenescence, chronic inflammation, and post-viral immune recovery without treating association as proof of causation.

## Epidemiological evidence for post-COVID HZ

Several large studies support an association between COVID-19 and subsequent HZ, although the magnitude should not be overstated. In a matched retrospective cohort of adults aged 50 years or older in the United States, COVID-19 diagnosis was associated with a 15% higher risk of HZ (adjusted incidence rate ratio [aIRR] 1.15, 95% confidence interval [CI] 1.07–1.24). The association was stronger among patients hospitalized for COVID-19 (aIRR 1.21, 95% CI 1.03–1.41) (Bhavsar et al., 2022). A population-based study from Spain that included more than four million adults similarly found increased HZ risk after laboratory-confirmed SARS-CoV-2 infection (adjusted relative risk 1.19, 95% CI 1.09–1.29), with higher estimates among hospitalized patients (Correcher-Martínez et al., 2024).

Longer follow-up also suggests a post-acute association. In a TriNetX propensity-matched study, individuals recovering from COVID-19 had an increased one-year risk of incident HZ (hazard ratio [HR] 1.59, 95% CI 1.49–1.69), with increased risks reported for herpes zoster ophthalmicus and disseminated HZ (Chen et al., 2023). A systematic review and meta-analysis likewise found an overall increase in HZ occurrence or recurrence after COVID-19, although pooled estimates varied with study design, population, and outcome definition (Wang et al., 2024). The consistency of direction supports an association, but variation in effect size and follow-up precludes a single causal estimate.

The timing of risk may be mechanistically informative. In a Japanese self-controlled case series, the incidence rate ratio for HZ was highest during weeks 1–2 after COVID-19 diagnosis (IRR 5.1, 95% CI 3.9–6.6) and remained elevated, although lower, during weeks 3–4 and 5–6 (Mizuno et al., 2025). This early peak is compatible with a transient reduction in cellular immune reserve during acute infection. A longer post-acute signal in other studies may reflect residual inflammation, treatment exposure, delayed immune recovery, or a combination of these factors.

Higher relative estimates after hospitalization or severe COVID-19 were reported in the United States and Spanish cohorts (Bhavsar et al., 2022; Correcher-Martínez et al., 2024), whereas the Japanese self-controlled study localized the strongest association to the first two weeks after diagnosis (Mizuno et al., 2025). These patterns favor effect modification over a uniform post-infectious complication. Interpretation therefore depends as much on host and clinical context as on the pooled magnitude of association.

Age-stratified evidence requires conservative interpretation. The United States cohort was restricted to adults aged 50 years or older (Bhavsar et al., 2022), whereas the Spanish cohort included all adults but did not show that younger adults had surpassed older adults in absolute HZ incidence (Correcher-Martínez et al., 2024). The TriNetX study reported a similar relative association across age subgroups, not an inversion of absolute incidence (Chen et al., 2023), and the meta-analysis found no clear age-group difference among reported post-infection cases (Wang et al., 2024). The proposed model therefore does not require an age inversion; it asks whether SARS-CoV-2 infection can move some susceptible hosts closer to an existing biological threshold.

Several dataset-level limitations affect interpretation. The United States claims cohort (Bhavsar et al., 2022), global TriNetX analysis (Chen et al., 2023), Spanish population cohort (Correcher-Martínez et al., 2024), and Japanese self-controlled study (Mizuno et al., 2025) identified HZ from coded clinical records rather than virological confirmation of VZV reactivation. The meta-analysis documented substantial heterogeneity across study designs, populations, and outcome definitions (Wang et al., 2024). Increased healthcare use after COVID-19, residual confounding by disease severity and comorbidity, and incomplete separation of corticosteroid or immunomodulatory treatment may also influence the observed estimates. These limitations constrain causal inference. Post-COVID HZ is better treated as a clinical signal arising when SARS-CoV-2 infection intersects with limited capacity for latency control.

## VZV latency and the immune-reserve threshold

VZV latency is an actively maintained state rather than simple viral silence. Evidence from human ganglia indicates restricted but organized latency-associated transcription (Kennedy et al., 2021). In neuronal models, the VLT-ORF63 fusion transcript can initiate broader viral gene expression during reactivation (Ouwendijk et al., 2020). Clinical HZ emerges when this balance shifts toward productive viral activity in a host unable to contain reactivation locally or systemically. The sensory ganglion is therefore both an anatomical reservoir and a site where viral transcriptional restraint, neuronal stress responses, satellite-cell biology, and systemic immunity converge.

For HZ, the principal antigen-specific immune correlate is VZV-specific adaptive cellular immunity (Weinberg and Levin, 2010). Anti-VZV antibodies document previous exposure, but antibody titer alone is not associated with HZ incidence or severity (Asada, 2019). NK-cell activity, type I interferon pathways, and local neuroimmune responses contribute to a broader innate antiviral environment but are not VZV-specific (Gerada et al., 2020). VZV-specific CD4+ and CD8+ effector and memory T cells mediate antigen-specific control of reactivation (Weinberg and Levin, 2010). Clinical studies commonly assess protection as composite VZV-specific cell-mediated immunity (Levin et al., 2003), while phase-specific profiling has demonstrated differences in CD4+ cytokine and cytotoxic responses between latent infection and HZ (Jin et al., 2023). Innate and adaptive responses can cooperate in containment, but the adaptive T-cell response provides the principal antigen-specific correlate of protection against HZ. A person may therefore remain seropositive while having insufficient VZV-specific cellular immune reserve to maintain latency under stress.

Aging remains the strongest population-level context for HZ, although chronological age is an imperfect proxy for biological reserve. Recent age-stratified work showed that VZV-specific cell-mediated immunity begins to decline from approximately age 40, whereas VZV DNA-based proxy measures of latent viral burden increase from approximately age 50; VZV-specific IgG did not show the same relationship (Son et al., 2025). These findings support a gradual loss of reserve rather than a discrete age threshold. Risk rises with age because immune reserve narrows, not because age alone is a sufficient mechanism.

Comorbidity data reinforce this interpretation. A recent United States retrospective cohort found that adults aged 30–39 years with asthma, chronic obstructive pulmonary disease, depression, diabetes, stress, or trauma had HZ incidence rates that exceeded those of immunocompetent adults aged 50–59 years without those comorbidities (Cohen et al., 2026). Although the study was not designed around COVID-19, it shows that chronic disease and stress can move susceptible hosts closer to the reactivation threshold before the usual high-risk age range. SARS-CoV-2 infection may add a further immune stressor to this background.

VZV is useful for studying latency failure because reactivation often produces a recognizable clinical syndrome. EBV, CMV, HHV-6, and other latent viruses may reactivate subclinically or require specialized assays, whereas VZV reactivation commonly presents as a dermatomal eruption. This clinical visibility is informative but also introduces ascertainment bias: patients with pain, rash, ophthalmic symptoms, or post-COVID follow-up are more likely to receive a diagnosis than those with unrecognized subclinical reactivation.

Immune reserve is best viewed as a margin of safety rather than a fixed numerical cutoff. A host with preserved VZV-specific cellular immunity may tolerate transient inflammatory stress without clinical reactivation. A host with depleted or poorly coordinated immunity may not. COVID-19 may contribute through an acute immune perturbation, prolonged inflammatory recovery, treatment-related immunosuppression, or combinations of these effects.

## Post-COVID immune disequilibrium and host modifiers

SARS-CoV-2 infection can create an immunological environment compatible with VZV reactivation. Severe COVID-19 has been associated with lymphopenia, impaired type I interferon responses, and inflammatory amplification (Hadjadj et al., 2020). Exhaustion-like T-cell states have been discussed across acute and post-acute COVID-19 (Chen-Camaño et al., 2025), while deep immunophenotyping has demonstrated persistent inflammation, altered T-cell subsets, and poorly coordinated adaptive responses in some individuals with long COVID (Yin et al., 2024). These abnormalities overlap with systems that normally restrain VZV.

These observations do not establish a VZV-specific bridge. Neither the severe-COVID interferon study (Hadjadj et al., 2020), the review of T-cell exhaustion (Chen-Camaño et al., 2025), nor the long-COVID immunophenotyping cohort (Yin et al., 2024) tested whether its immune signatures predicted HZ. Conversely, VZV research identifies cellular immunity (Weinberg and Levin, 2010) and innate antiviral pathways (Gerada et al., 2020) as components of latency control, but has not evaluated them longitudinally after COVID-19. Recognized immunosuppressive and comorbid conditions may further reduce reserve (Marra et al., 2020). In a susceptible host, even a temporary reduction in lymphocyte number or function may allow low-level viral activity to become symptomatic.

Interferon biology offers another plausible link. Severe COVID-19 can combine impaired type I interferon activity with exaggerated inflammation (Hadjadj et al., 2020). VZV can manipulate innate signaling (Gerada et al., 2020), and experimental work distinguishes an early IFN-α–IRF9 response that delays viral spread from a stronger IFN-γ–IRF1 axis that suppresses replication (Sen et al., 2018). These findings establish biological plausibility, but not a VZV-specific post-COVID mechanism. Longitudinal studies should measure interferon signatures together with VZV-specific T-cell responses and subclinical viral activity.

Host and treatment factors may substantially modify any infection-related effect. Diabetes, chronic pulmonary disease, chronic kidney disease, depression, psychological stress, trauma, autoimmune disease, malignancy, and HIV infection are associated with higher HZ risk (Marra et al., 2020). Systemic corticosteroids also increase HZ risk in a dose-related manner (Qian et al., 2021). These factors cluster in patients who are more likely to experience severe COVID-19, hospitalization, and immunomodulatory treatment. Separating pre-existing vulnerability and treatment exposure from infection-related effects is therefore essential.

**Figure 1:**
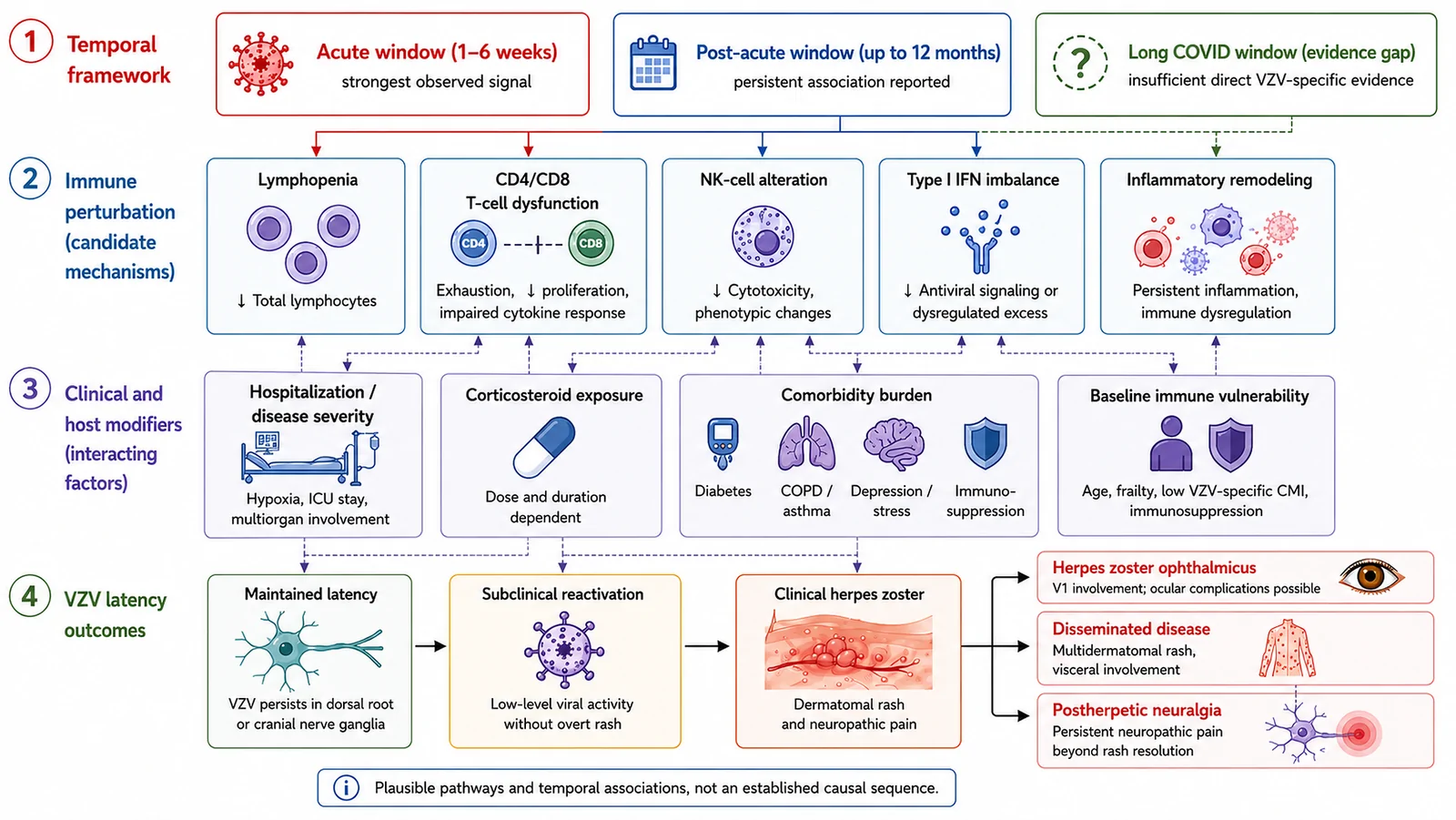
Temporal and mechanistic model of post-COVID VZV reactivation. SARS-CoV-2 infection may be followed by early and post-acute immune perturbations, including lymphopenia, CD4+ and CD8+ T-cell dysfunction, altered NK-cell activity, type I interferon imbalance, and inflammatory remodeling. These changes may interact with disease severity, hospitalization, corticosteroid exposure, comorbidity burden, and baseline immune vulnerability to reduce the immune reserve required to maintain VZV latency. The model summarizes plausible pathways and temporal associations rather than an established causal sequence. The figure was redrawn and finalized by the authors using Adobe Illustrator (Adobe Inc.).

**Figure 1** organizes timing, immune perturbations, host modifiers, and clinical outcomes without implying that every patient follows the same sequence. Acute lymphopenia may be most relevant to early cases, persistent inflammation to post-acute cases, corticosteroid exposure to hospitalized patients, and comorbidity burden to hosts whose reserve was already low. HZ is also not a single endpoint: uncomplicated dermatomal HZ, herpes zoster ophthalmicus, disseminated disease, and postherpetic neuralgia may represent different degrees or consequences of impaired immune control.

Long COVID is relevant as a research setting rather than a proven causal context. Deep immune profiling has shown that some individuals with long COVID have systemic inflammation, altered T-cell subset distributions, exhausted SARS-CoV-2-specific CD8+ T cells, and poorly coordinated adaptive responses months after infection (Yin et al., 2024). A prospective study associated selected EBV and CMV measures with distinct long-COVID phenotypes (Peluso et al., 2023). A broader herpesvirus review concluded that direct VZV-specific evidence remains limited (Gáspár et al., 2025). In this setting, HZ could be a marker, mediator, or bystander. Distinguishing among these possibilities requires cohorts that define long COVID rigorously and measure both HZ outcomes and VZV-specific immunity. The possibility that COVID-19 accelerates aspects of immunosenescence and neuroimmune aging provides additional biological context, but remains a hypothesis (Müller and Di Benedetto, 2024).

Recent database studies are compatible with, but do not prove, a marker hypothesis. One TriNetX analysis linked HZ after COVID-19 with later cardiorenal outcomes (Lu et al., 2025); a separate analysis reported increased risks of uveitis and optic neuritis (Lin et al., 2026). These findings do not show that VZV drives those outcomes. Clinically apparent HZ may instead occur within a subgroup characterized by broader immune, vascular, metabolic, or inflammatory vulnerability after COVID-19. HZ should not itself be regarded as identifying or defining that subgroup.

## Clinical implications and research priorities

Clinically, the threshold model supports focused vigilance rather than broad screening. It does not justify routine VZV testing after every SARS-CoV-2 infection or redefine HZ as an inevitable COVID-19 complication. Older adults, immunocompromised patients, and adults with diabetes, chronic pulmonary disease, chronic kidney disease, recent corticosteroid exposure, or marked psychological stress may have less reserve for latency control. In these groups, unilateral neuropathic pain, a dermatomal rash, ophthalmic symptoms, or atypical disseminated lesions after COVID-19 warrant prompt recognition and treatment according to existing clinical standards.

Although vaccination is not the main focus of this review, childhood varicella vaccination policy could contribute to between-study heterogeneity. The principal post-COVID evidence came from a United States claims cohort (Bhavsar et al., 2022), a global TriNetX cohort (Chen et al., 2023), a Spanish population cohort (Correcher-Martínez et al., 2024), a Japanese self-controlled study (Mizuno et al., 2025), and a meta-analysis (Wang et al., 2024). None reported a comparison stratified by national childhood varicella vaccination policy, and individual childhood vaccination history was not consistently available. The United States cohort was restricted to adults aged 50 years or older (Bhavsar et al., 2022), most of whom were born before routine childhood varicella vaccination; national policy is therefore an imperfect proxy for personal vaccination status. A United States population analysis did not attribute increasing adult HZ incidence to the childhood program (Hales et al., 2013). Separately, post-vaccine birth cohorts showed declining HZ incidence (Leung et al., 2022). Future studies should record individual varicella vaccination history and account for national policy, HZ vaccine uptake, population age structure, calendar period, healthcare access, and case ascertainment.

Future studies should account for baseline differences in immune reserve rather than treating COVID-positive and COVID-negative groups as biologically equivalent. Analyses should be stratified by age, comorbidity, acute disease severity, hospitalization, corticosteroid or immunomodulatory treatment, vaccination status, and healthcare use. Whenever feasible, clinical outcomes should be paired with VZV-specific cellular immune assays, inflammatory markers, and measures of latent or subclinical herpesvirus activity, including VZV DNA detection, serological dynamics, or longitudinal changes in VZV-specific cellular immunity. Such designs would help distinguish direct immune disruption from pre-existing vulnerability, treatment-related immunosuppression, and combinations of these factors.

Age terminology also requires care. HZ in adults younger than the usual vaccine-eligible age range should not be labeled simply as “younger shingles”. That phrase conflates absolute population incidence with earlier clinical expression in a susceptible subgroup. Age remains a major determinant of risk, but comorbidity, stress, treatment, and post-COVID immune disequilibrium can shorten the distance between a given host and the VZV reactivation threshold.

A practical study design would enroll patients at defined intervals after SARS-CoV-2 infection, document acute disease severity and treatment, measure VZV-specific T-cell responses, and track both clinical HZ and subclinical herpesvirus activity. Analyses should be age-stratified but not age-restricted. This approach would test whether immune reserve, rather than chronological age alone, helps explain why some hosts develop HZ earlier than expected after COVID-19.

## Discussion: threshold lowering, not age inversion

**Figure 2:**
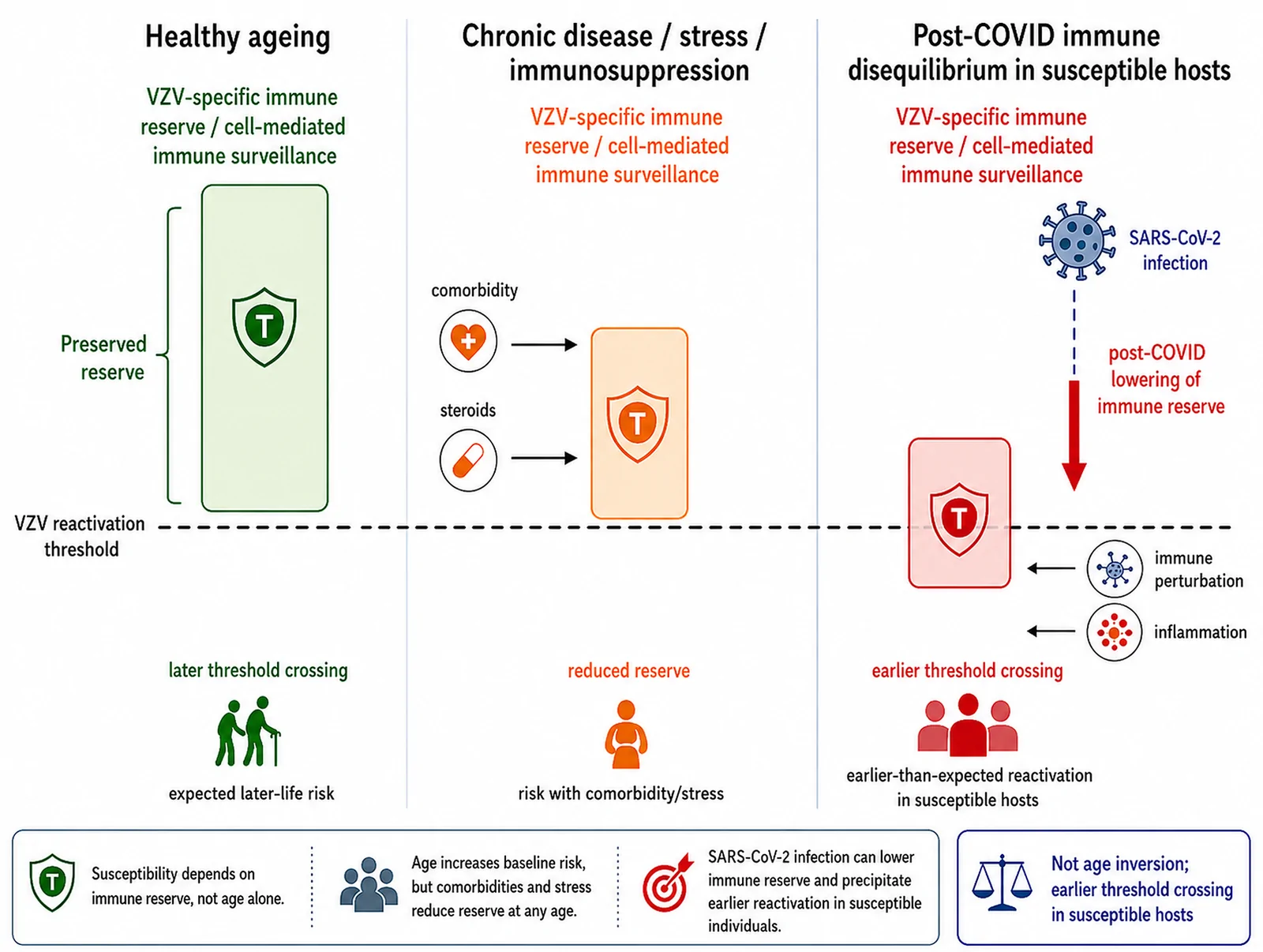
Lowered VZV reactivation threshold model. The model illustrates how VZV-specific immune reserve may remain preserved during healthy aging, become narrowed by chronic disease, stress, or immunosuppression, and be further reduced in susceptible hosts after SARS-CoV-2 infection. This framework does not imply age inversion or higher absolute HZ incidence in younger adults. Instead, it proposes earlier threshold crossing in susceptible hosts whose immune reserve has been lowered by comorbidity, inflammation, treatment exposure, or post-COVID immune disequilibrium. The figure was redrawn and finalized by the authors using Adobe Illustrator (Adobe Inc.).

The threshold concept permits earlier-than-expected HZ after COVID-19 to be described without implying that younger adults have overtaken older adults in absolute incidence. The evidence supports a narrower inference: SARS-CoV-2 infection may reduce immune reserve sufficiently for some susceptible hosts to cross the VZV reactivation threshold earlier than chronological age alone would predict (**Figure 2**).

Under this framework, HZ occurring after SARS-CoV-2 infection is not taken as proof of causation, and selected cases are not construed as evidence of “young shingles,” an epidemiological shift that has not been demonstrated. Age, baseline immune status, comorbidity, treatment exposure, and acute infection are considered within the same explanatory framework.

This framework yields testable predictions. If threshold lowering is correct, post-COVID HZ should not be predicted by age, infection status, or antibody status alone. Prediction should improve when VZV-specific IFN-γ-producing T-cell responses, markers of latent or subclinical herpesvirus activity, inflammatory markers, comorbidity, corticosteroid exposure, and acute disease severity are considered together. Prospective studies should combine age-stratified follow-up with careful HZ phenotyping and immunological measurements that distinguish uncomplicated dermatomal HZ, herpes zoster ophthalmicus, disseminated disease, neurological complications, and postherpetic neuralgia.

Several limitations remain. The United States claims study (Bhavsar et al., 2022), global TriNetX analysis (Chen et al., 2023), Spanish population cohort (Correcher-Martínez et al., 2024), and Japanese self-controlled study (Mizuno et al., 2025) relied on routinely coded clinical data and did not directly measure VZV-specific T-cell immunity. The available meta-analysis also shows substantial methodological heterogeneity (Wang et al., 2024). The main long-COVID immunophenotyping study did not include clinical VZV reactivation as a predefined outcome (Yin et al., 2024). A prospective coinfection study concentrated on EBV and CMV measures (Peluso et al., 2023), while a broader review found little direct VZV-specific evidence (Gáspár et al., 2025). Treatment effects remain difficult to separate from acute disease severity in observational data. The same post-COVID cohorts did not consistently capture national childhood varicella vaccination policy or individual vaccination history. These constraints should temper causal interpretation of the current evidence.

The available evidence supports a modest but reproducible association between SARS-CoV-2 infection and HZ, particularly after severe disease and during early post-infection windows. Biological plausibility rests on cellular immune perturbation, interferon imbalance, inflammation, comorbidity, and treatment-related immunosuppression. The immediate research priority is to determine whether these factors measurably reduce VZV-specific cellular immunity and whether that reduction predicts earlier clinical reactivation. Post-COVID HZ may therefore serve as a visible indicator of impaired herpesvirus latency control, but not as proof of a uniform causal pathway.
